# Measuring the Transmembrane Registration of Lipid Domains in Droplet Interface Bilayers through Tensiometry

**DOI:** 10.1021/acs.langmuir.4c00958

**Published:** 2024-05-16

**Authors:** Braydon G. Segars, Michelle Makhoul-Mansour, Joyce Beyrouthy, Eric C. Freeman

**Affiliations:** † School of Environmental, Civil, Agricultural, and Mechanical Engineering, University of Georgia, 110 Riverbend Road, Athens, Georgia 30605, United States; ‡ Mechanical, Agricultural, Biomedical, and Environmental Engineering Department, Tickle College of Engineering, University of Tennessee Knoxville, 1512 Middle Dr., Knoxville, Tennessee 37916, United States

## Abstract

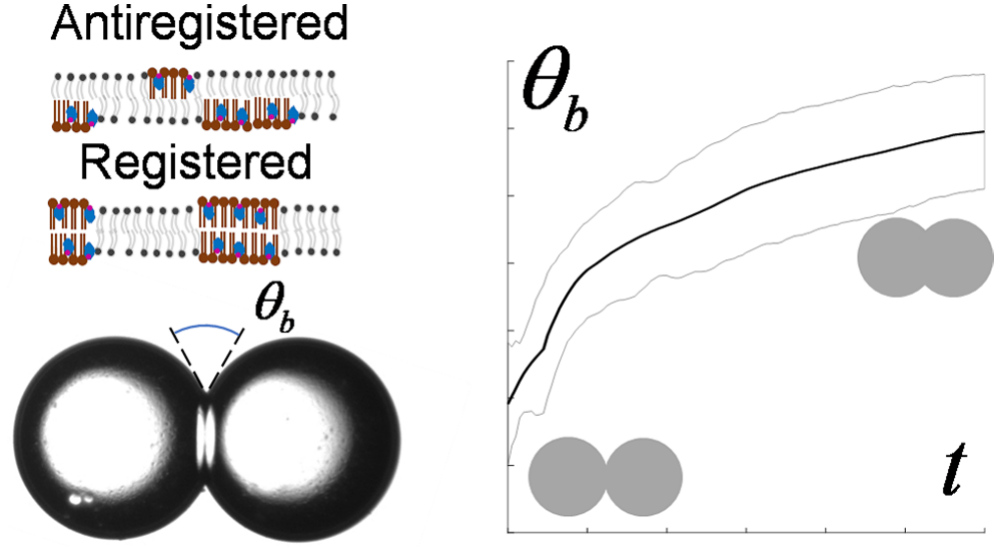

Diverse collections of lipids self-assemble into domains within biological membranes, and these domains are typically organized in both the transverse and lateral directions of the membrane. The ability of the membrane to link these domains across the membrane’s interior grants cells control over features on the external cellular surface. Numerous hypothesized factors drive the cross-membrane (or transverse) coupling of lipid domains. In this work we seek to isolate these transverse lipid–lipid influences in a simple model system using droplet interface bilayers (DIBs) to better understand the associated mechanics. DIBs enable symmetric and asymmetric combinations of domain-forming lipid mixtures within a model bilayer, and the evolving energetics of the membrane may be tracked using drop-shape analysis. We find that symmetric distributions of domain-forming lipids produce long-lasting, gradual shifts in the DIB membrane energetics that are not observed in asymmetric distributions of the lipids where the domain-forming lipids are only within one leaflet. The approach selected for this work provides experimental measurement of the mismatch penalty associated with antiregistered lipid domains as well as measurements of the influence of rafts on DIB behaviors with suggestions for their future use as a model platform.

## Introduction

### Lipid Membranes—Function and Organization

Cellular organisms contain collections of semipermeable membranes, delineating the cytoplasm from the extracellular fluid.^1^ Serving as a thin, enclosing sheet, the cell membrane facilitates compartmentalization and controlled exchanges,^2^ fostering the formation of subcellular organelles and mediating biochemical signals.^2^ Lipid is a broad category encompassing molecules such as phospholipids and cholesterol, which are the primary components of the cell membrane. Lipids form the standard membrane bilayer structure, consisting of two opposing leaflets.^2^ The diversity of these lipids enables the formation of subdomains wherein the lipids spontaneously produce ordered substructures given their differing properties.^3^

Cellular membranes naturally arrange their lipid constituents into distinct domains with varying properties.^4,5^ Different lipids possess distinct melting temperatures which determine their behavior within the membrane. Above this melting temperature they exist in a disordered state marked by disordered acyl chains and fast lateral diffusion.^6^ Below this melting temperature, they exist in a gel state where they are highly ordered.^6^ When membranes contain lipids with different melting temperatures, the membrane often exhibits lateral organization, forming ordered (L_o_) and disordered (L_d_) domains. These mixtures are analogous to ordered icebergs (domains) contained within a disordered sea.^6^

**Figure 1:**
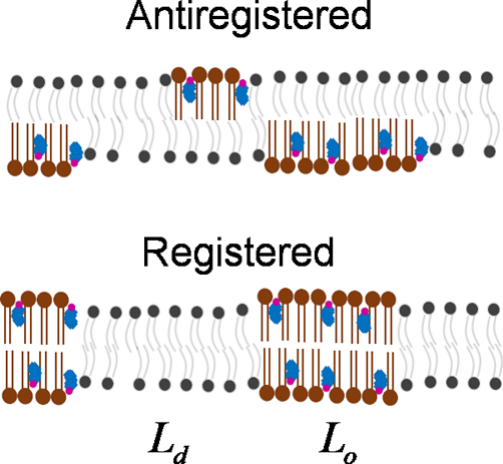
Mixtures of lipids with varying transition temperatures frequently produce domains within the membrane of ordered (L_o_) and disordered (L_d_) regions. These domains are frequently collocated across the membrane interior, favoring registration (mirrored composition in the two leaflets) vs antiregistration (varying compositions).

The ordered domains are frequently collocated across the membrane.^7^ The transverse coupling of these domains has been investigated as functions of membrane undulation,^8^ cholesterol-driven registration,^9^ interdigitation of the lipid tails,^10^ and line/leaflet tensions.^9^ Domains that are mirrored across the lipid membrane are said to be registered, while domains that are asymmetric within the membrane are reported as antiregistered (Figure 1). The registration of these domains across the membrane has been a topic of interest, as they enable cross-membrane control of cellular surface elements from within the cellular interior.^11^ Membrane elements may be grouped or collocated through the organization of the lipids to which they are attached,^12^ such as specific lipid–protein interactions^13−17^ and hydrophobic matches or mismatches.^18^

Registration is often summarized through the energy-per-area difference between registered and antiregistered domains,^11,19^ encompassing each of the terms associated with domain coupling listed previously. The value of the energy mismatch varies based on the specific lipid compositions under investigation and typically ranges between 0.01 and 1 *k*_B_*T*/nm^2^ (approximately 0.005 to 0.5 mN/m).^9,11,20−23^ Ultimately a membrane possessing registered domains is expected to be energetically favorable relative to an antiregistered membrane, and once registration begins it will continue as the membrane gradually seeks the most favorable arrangement of lipids possible within the constraints of the membrane. As a result, we expect to see a gradual reduction in the cost of formation of a bilayer membrane as the domains in the two leaflets continue to register together over time.

### Model Membrane Systems

Investigating the specific interactions between individual lipids that govern domain formation in vivo is often challenging.^24,25^ Instead, simplified model systems are frequently used to recreate the desired lipid–lipid interactions in a controlled environment, isolating the phenomenon of interest. Model membranes, *in vitro* recreations of lipid membranes, have been essential for studying lipid domains.^5^ There are many different techniques for forming lipid membranes such as liposomes,^26,27^ Langmuir monolayers,^28^ and supported lipid membranes.^29^ Each of these models relies on self-assembly principles for their manufacture, and the selected method for assembly often influences the properties of the produced membranes as well as the available methods for membrane characterization.^30^

**Figure 2:**
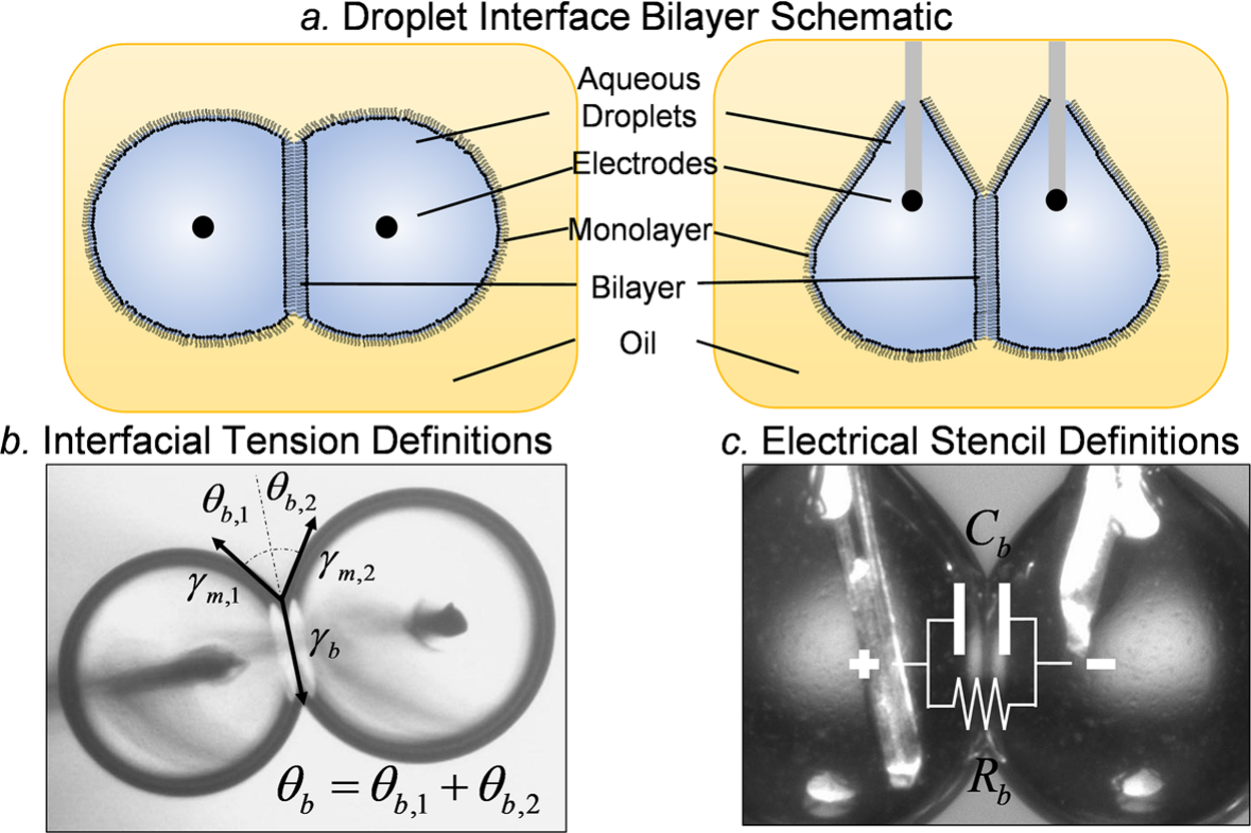
The DIB technique is used to form model lipid membranes. (a) Aqueous droplets are dispersed onto agarose-tipped silver/silver chloride electrodes, where lipids unfold at the oil–water interface to form the lipid monolayer. The droplets are brought together to form the lipid bilayer, and images are taken from the bottom and side views for analysis. (b) The sum of the surface tensions predicts the apparent bilayer tension based on the monolayer tension and the measured contact angle. (c) The membrane can be electrically approximated as a capacitor and a resistor in parallel.

The droplet interface bilayer (DIB, Figure 2) technique was selected for this study.^1^ In this approach, aqueous droplets are submerged in an oil reservoir with dispersed lipids in either phase. The lipids then form a self-assembled lipid monolayer around the droplets at the oil–water interfaces. Lipid bilayers are formed when the lipid-coated droplets are brought into contact and the oil separating the monolayers is dispersed.^31^ The dimensions of the droplets and lipid bilayers are controlled by the equilibrium of tensions (Figure 2b), minimizing the total interfacial energy of the adhered droplet pair and linking the tensions to the measured contact angle.^1^ The DIB membranes do exhibit higher tensions than their physiological counterparts (∼1–2 mN/m for DIB membranes^32^ compared against ∼0.2 mN/m for cell membranes^33^), but DIBs provide visual estimations of the membrane properties through drop-shape analysis^1,30,34,35^ which will be used in this study to track domain registration between the leaflets of the bilayer membrane.

### Proposed Approach

This research looks to further investigate the effects of lipid domain registration on the properties of the membrane. This is accomplished through a combination of pendant drop tensiometry with drop shape analysis, using a 1:1:1 molecular ratio of 1,2-diphytanoyl-*sn*-glycero-3-phosphocholine (DPhPC, low melting temperature), cholesterol, and brain sphingomyelin (bSM, high melting temperature). DPhPC provides robust model membranes while excluding cholesterol where feasible as indicated monolayer tension measurements,^1,36^ and bSM and cholesterol combined produce ordered domains. This mixture allows for the study of domain influences within the DIB membrane and has been studied previously in droplet–hydrogel bilayers.^4^

Here we propose a simple model for membrane energetics. The bilayer tension γ_b_ may be expressed as a base tension γ_b,0_ (assumed at equilibrium or the best packed configuration feasible) combined with the mismatch energy γ_anti_ associated with antiregistration of the leaflets encompassing the interdigitation, undulation, electrostatics, and cholesterol-driven contributions hypothesized to drive registration as discussed previously (eq 1).
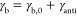
1This bilayer tension may be experimentally estimated using contact angle analysis with DIBs as discussed in previous works.^1,30,34,35^ At equilibrium, the tensions will be balanced at the intersection of the bilayer and monolayers (Figure 2b). Measurements of the contact angles  may be combined with measurements of the monolayer tension  to estimate the current bilayer tensions. Monolayer tension may be obtained through pendant drop tensiometry.^37^
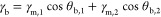
2Calculation of the bilayer tension may be used to estimate the energy of adhesion of the membrane, or ε.^1^ This represents the reduction in energy associated with replacing two monolayer interfaces with a single bilayer interface and may be calculated as^38^
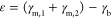
3These values will be combined with electrophysiology, measuring the total membrane capacitance and conductance over time, assuming the membrane may be approximated as a resistor in parallel with a capacitor (Figure 2c). These values may be converted into measurements of the specific capacitance by dividing the measured capacitance (*C*_b_) by the membrane area (*A*_b_), which may be linked to the membrane thickness *h* and relative permittivity ε_r_ as shown in eq 4.
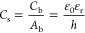
4This dielectric thickness is linked to trapped solvent within the membrane combined with the base membrane dimensions.^36,39^ Asymmetric membranes may be produced by adjusting the lipid composition of the two droplets,^40,41^ and it is possible to form the DIB either at elevated temperatures prior to domain formation or immediately after domains are formed in the two leaflets separated by the oil^42^ to control the initiation of lipid domains in the leaflets. Measurements of the membrane tension and capacitance will be used to track gradual changes in the membrane properties as domains are formed and registered.

## Experimental Section

### Materials

Three lipid in solutions are prepared (lipids dispersed in the aqueous phases). These are mixtures of DPhPC:bSM:cholesterol at varying molar ratios, either 1:0:0, 0:1:1, or 1:1:1. These mixtures were selected for stability, comparison against past results in DIBs through the inclusion of bSM:cholesterol in a DPhPC membrane, and their previously resolved propensity for domain formation in similar model systems.^4^ All lipids were obtained in chloroform from Avanti Polar Lipids. Mixtures were prepared according to the desired ratios; then the chloroform in the solutions was initially evaporated using a gentle flow of argon gas and then left in a vacuum chamber overnight to ensure complete evaporation. The resulting lipid film is then hydrated with a buffer solution to produce 2 mg/mL final concentrations of the lipids and put through six freeze–thaw cycles once hydrated. The buffer solution consists of 250 mM of potassium chloride (KCl, ≥99.1%, Sigma-Aldrich) and 10 mM of 3-(*N*-morpholino)propanesulfonic acid (MOPS, ≥99.5%, Sigma-Aldrich).

The stock solutions are kept in a freezer at −4 °C. Solutions are thawed and then sonicated in multiple steps. First the solution is bath sonicated (Elmasonic S100h) at elevated temperatures above the transition temperatures for 45 min at 50 °C and then probe sonicated for four 2 min cycles before use, allowing for 2 min between sonication steps to return to room temperature (QSONICA Q5 probe tip ultrasonicator). Experimental observations indicate that bath sonication at elevated temperature is essential for ensuring even distributions of the lipid mixture within the unilamellar vesicles. Hexadecane (99%, Sigma-Aldrich) is used as the oil in all experiments aside from final experiments where tetradecane (99%, Sigma-Aldrich) is used for comparisons.

### Methods

#### Pendant Drop Tensiometry

**Figure 3:**
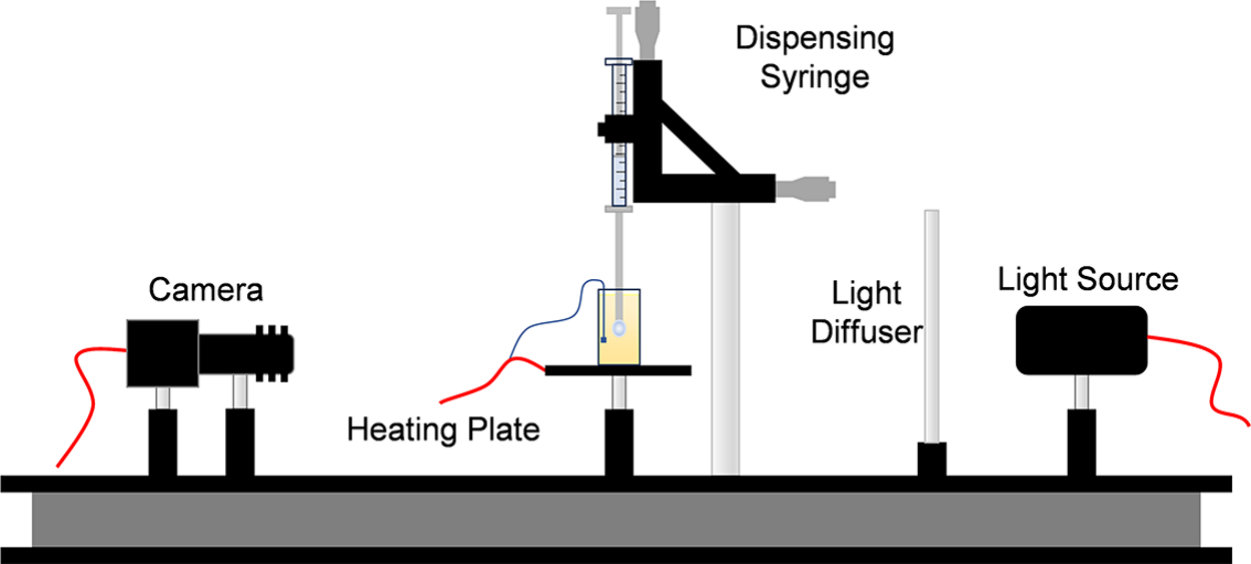
Experimental setup used for pendant drop tensiometry. The lipid solution is dispersed using a syringe with a stainless-steel needle into a cuvette filled with hexadecane mounted on a heating plate. A zoom lens camera takes images of the droplet from the side, capturing the pendant shape of the droplet. Modified with permission from ref (43). Copyright 2019 Royal Society of Chemistry.

Figure 3 shows the experimental setup used for pendant drop tensiometry described in previous works.^1,43^ Aqueous droplets are suspended from the tip of a stainless-steel needle within a cuvette filled with oil using a gastight syringe. The cuvette is fixed on top of a heating plate (Cell MicroControls TC2BIP) with a temperature sensor positioned in the oil adjacent to the suspended droplet. A zoom lens camera (DCU223M, 6.5× zoom lens with 0.7–4.5 magnification range, Thorlabs) is used to record video of the droplet over time. The frames are analyzed using OpenDrop pendant drop analysis software.^44^

Three mixtures of DPhPC:bSM:cholesterol are investigated (1:0:0, 1:1:1, and 0:1:1) using pendant drop tensiometry to see the effects of temperature on monolayer formation due to lipid domain formation. Two heating cases are examined. For case 1 the droplet is suspended at room temperature and given an hour for monolayer formation and to reach equilibrium. For case 2 the droplet is suspended at 50 °C to ensure a homogeneous distribution of the lipids, given 10 min for equilibrium tension; then the heating plate is turned off, and the droplet is allowed to cool to room temperature and given 1 h to reach a new equilibrium. Frames from the last 2 min of the recording are taken and analyzed using the open-source software OpenDrop^44^ to calculate the interfacial tension at equilibrium for each case.

To limit evaporation of the suspended droplets over the course of the experiments, the top of the cuvette was sealed, and excess droplets of the aqueous solution were deposited at the bottom of the cuvette containing the oil. However, cooling the droplet down from elevated temperatures required adjustments to the droplet volume due to trapped air in the syringes. All precautions were taken to eliminate these, but droplet motion was still observed during cooling due to the low interfacial tensions (∼0.5–1.0 mN/m) and small droplet volumes required for the low tensions. Additional fluid was added to the suspended droplet to ensure the droplet maintained a pendant shape when necessary. These additions appeared to influence the equilibrium tension minutely, as the newly formed monolayer area was established at an elevated temperature and the droplet inflation temporarily increased the area per lipid.

#### Contact Angle Analysis

**Figure 4:**
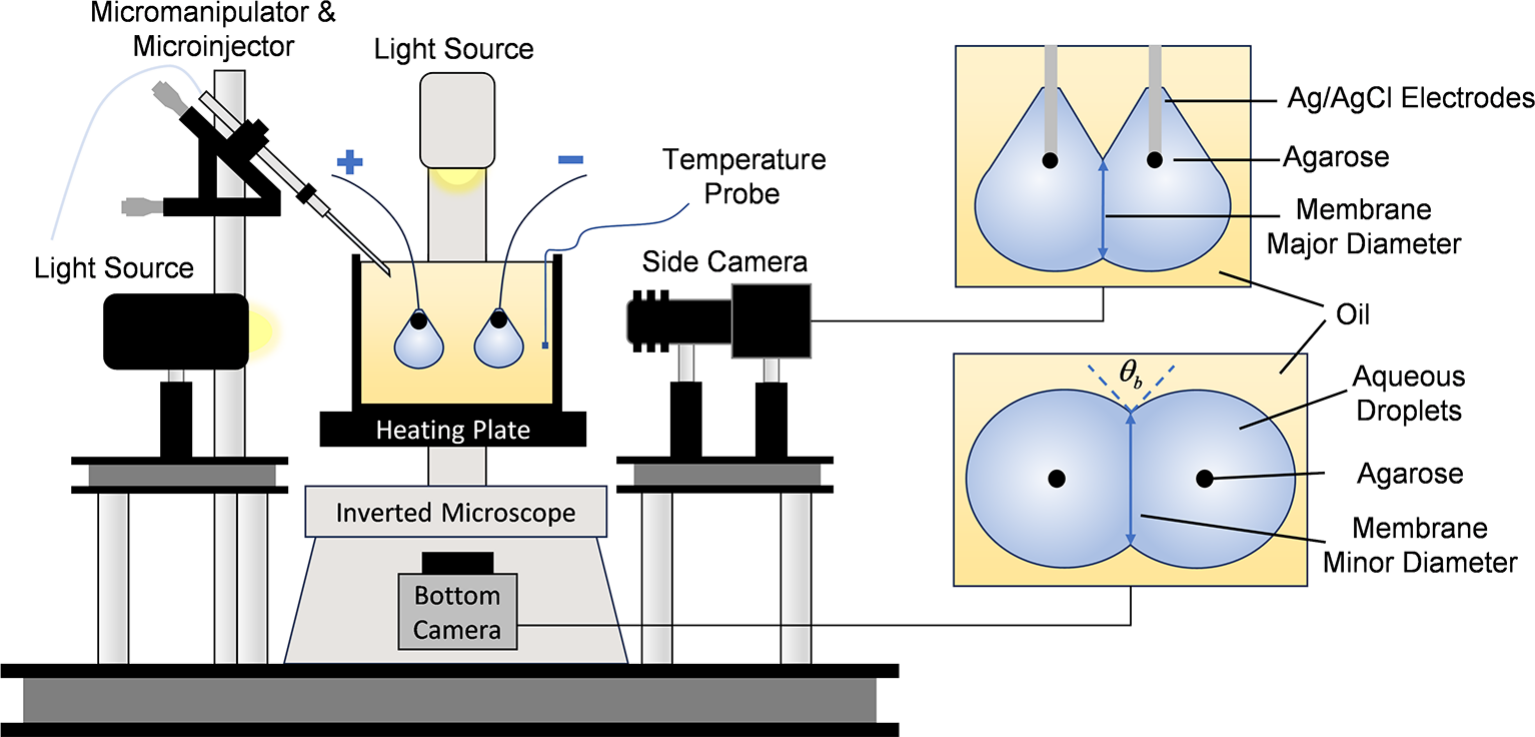
Experimental setup used for the creation and characterization of DIBs. The droplets are submerged in an acrylic glass dish with hexadecane and connected to electrophysiology equipment. An inverted microscope and a zoom lens camera are used to enable simultaneous measurements of the bilayer area and contact angle.

Figure 4 shows the experimental setup used for the contact angle experiments used in previous works.^1^ This setup allows for the accurate characterization of DIBs with simultaneous measurements of membrane area (*A*_m_) and contact angles between the droplets (Θ_b_). Aqueous droplets are deposited onto the two silver/silver chloride (Ag/AgCl) electrodes (50 μm annealed silver wire, GoodFellow), which are submerged into an acrylic glass dish filled with oil, typically hexadecane. In this work the acrylic dish is placed on top of a heating plate with the temperature sensor placed in the oil. The tips of the Ag/AgCl electrodes are coated with an agarose gel (2 mass % A9539, Sigma-Aldrich) to aid in droplet–electrode adhesion. The electrodes are connected to an Axopatch 200B patch-clamp amplifier and a Digidata 1440 data acquisition system (Molecular Devices). A prescribed voltage is maintained between the electrodes, and the current necessary to maintain this voltage is recorded. Voltage-clamp mode (whole cell β = 1) is used at a 5 kHz sampling frequency with a low pass filter of 1 kHz (using the embedded low-pass Bessel filter −80 dB/decade). Before each experiment, the pipet offset is compensated by coalescing the droplets and adjusting the offset to *V* = 0 in the current-clamp mode. Residual electrode capacitance is eliminated using the patch clamp amplifier’s built-in whole-cell capacitance compensation before membrane formation. A side-view camera (DCU223M, 6.5× zoom lens with 0.7–4.5 magnification range, Thorlabs, Newton, NJ) is used to record video to analyze the side-view diameter of the membrane. A bottom-view camera (DCC1645C, Thorlabs, Newton, NJ) is used to record video to analyze the bottom-view diameter of the membrane and is set at a fixed zoom through an inverted microscope (Axiovert-Zeiss).

**Figure 5:**
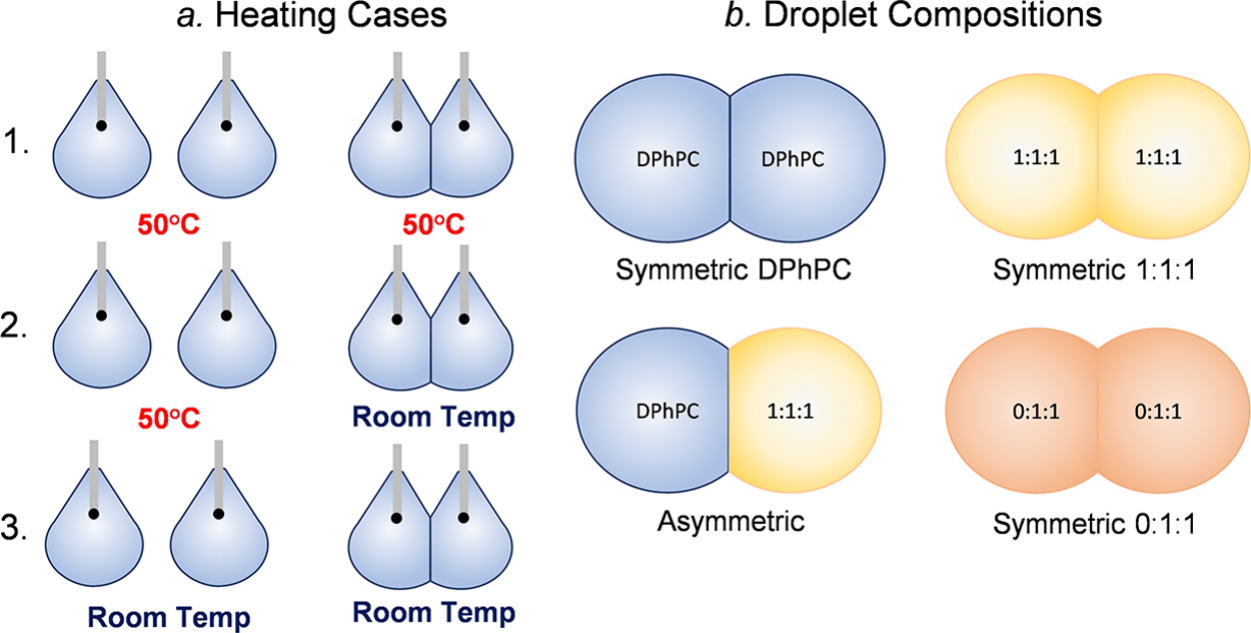
(a) Three heating cases are examined; (1) monolayer and bilayer formed at 50 °C, (2) monolayer formed at 50 °C and bilayer formed at room temperature after cooling, and (3) monolayer and bilayer formed at room temperature. (b) Four droplet compositions will be examined: (1) symmetric 1:1:1, (2) symmetric 1:0:0, (3) asymmetric 1:0:0 and 1:1:1, and (4) symmetric 0:1:1.

The droplet compositions consist of symmetric droplets of 1:1:1 (DPhPC:bSM:cholesterol, molar ratios) solution, symmetric droplets of 0:1:1 solutions, asymmetric droplets of 1:0:0 and 1:1:1 solution, and symmetric droplets of 1:0:0 as shown in Figure 5b. Two sets of experiments were conducted. The first experiments relied on contact angle measurements alone, depositing the droplets within the oil and manipulating them together to form the membrane. Micrographs were taken immediately before heating once the membrane reached equilibrium and then after cooling. Changes in the contact angle between the droplets were recorded.

Later experiments examined the evolution of the specific capacitance and contact angle over time with varying conditions. A summary of the heating used for each composition is shown in Figure 5a. For case 1, the droplets were heated past the transition temperature, connected to form a membrane above the transition temperature to ensure domains were not initially present, and then cooled to room temperature. In case 2 droplets were heated past the transition temperature, cooled, and then connected to form a membrane. This produced lipid domains in the monolayers prior to membrane formation. In case 3, no heat was applied. This last case was primarily used for membranes without domain-forming lipids (1:0:0 composition), but results were obtained for the symmetric 1:1:1 composition as well (Figure S3).

In these experiments the lipid solution is injected directly onto the electrodes submerged in the oil. A sinusoidal voltage input of 10 mV and 40 Hz is applied using the patch-clamp amplifier to measure the membrane capacitance, and video recordings were taken of the side view and bottom view to measure both radii of the membrane (Figure 4). The recordings are started when lipid bilayer formation begins and are typically conducted for 2 h to observe changes in the membrane characteristics over time. Frames are taken from the bottom camera video and analyzed using MATLAB to find the contact angle and membrane minor diameter. The imfindcircles() command is used to locate the center and radius of each droplet and the distance between the centers of the droplets, and their dimensions are used to find the two overlapping points that define the membrane minor axis. These lines are plotted on the input image and exported to ensure consistency. Frames are taken from the side camera video and analyzed by hand to find the membrane major diameter. If the droplets are distorted causing errors in interpretation, then the experiment is repeated. The membrane dimensions are used to convert the membrane capacitance into specific capacitance *C*_s_, or capacitance per area. When plotting results for the contact angle θ_b_ or specific capacitance *C*_s_ and averaging with respect to time across multiple samples, the data were smoothed in MATLAB to eliminate noise (due to variance between frames in the image analysis) using a moving average approach with a fixed window dimension of 125 frames (approximately 10 min).

Cases where the membrane was formed at elevated temperatures were cooled after the membrane had been established for 5 min. This cooling process introduced complications with convective flow of the oil and electrode drifting; these were handled by manually manipulating the droplet positions to keep them relatively consistent throughout the cooling process. After approximately 20 min, the droplets reached room temperature and manual manipulation was no longer required. In cases where the results are reported as energy of adhesion or bilayer tension, these values are reported beginning at 20 min as they rely on the monolayer tension values obtained at room temperature.

## Results and Discussion

### Pendant Drop Tensiometry Results

**Figure 6:**
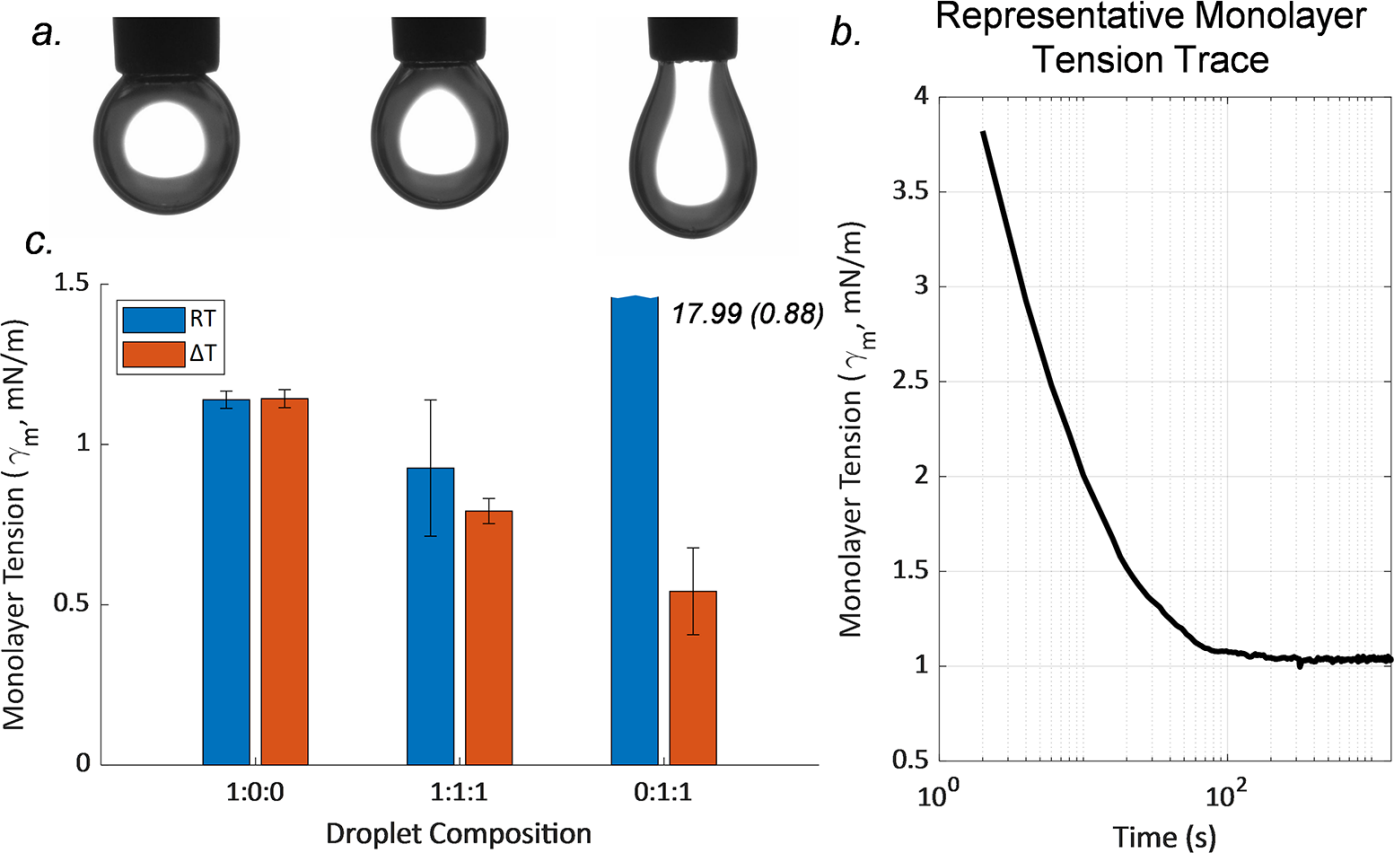
The pendant drop technique used to measure the monolayer surface tension of the aqueous lipid solution. (a) Representative experimental images of monolayer tension change over time. (b) Representative monolayer tension trace over time. (c) Pendant drop tensiometry results for droplets with 1:0:0 (*n* = 5 for RT and Δ*T*), 0:1:1 (*n* = 4 for RT, *n* = 5 for Δ*T*), and 0:1:1 (*n* = 4 for RT, *n* = 3 for Δ*T*) DPhPC:bSM:cholesterol mixtures for two heating cases. Case 1 is done at room temperature (RT), and case 2 is deposited at 50 °C and cooled to room temperature (Δ*T*).

Figure 6 displays the averages and the standard deviations in monolayer tensions for all six cases. For the 1:1:1 solution forming the monolayer at room temperature resulted in an interfacial tension of 0.93 ± 0.18 mN/m. Forming the monolayer at 50 °C and cooling the droplet to room temperature resulted in an interfacial tension of 0.79 ± 0.04 mN/m. This suggests that while it is feasible to form a 1:1:1 membrane without heating, the results are more consistent using an initial heating step as given by the lowered deviation in the results. Supporting this observation, the 0:1:1 solution was unable to form a low-tension monolayer (17.99 ± 0.88 mN/m tension) without heating; however, when heated past the transition temperature and then cooled, the bSM and cholesterol are able to form domains and create a low-tension monolayer (0.542 ± 0.11 mN/m). 1:0:0 exhibited a negligible temperature dependence for monolayer surface tension. Forming the 1:0:0 monolayer at room temperature or at 50 °C both resulted in an interfacial tension of 1.14 ± 0.03 mN/m.

Interfacial tension results from pendant drop tensiometry show that heating the 1:1:1 solution ensures proper monolayer formation. This confirms previous research on complex lipid mixtures in DIBs, showing that increased temperature past the transition melting temperature is often necessary for complex monolayer formation at the droplet surfaces.^42^ These experiments were conducted for an hour, and cases where the droplet separated from the needle tip were not included in the final results. The final monolayer tensions were stable, and minimal changes were observed over time as shown in Figure 6b, suggesting that the domain formation did not significantly influence the monolayer tensions.

### Contact Angle Results

**Figure 7:**
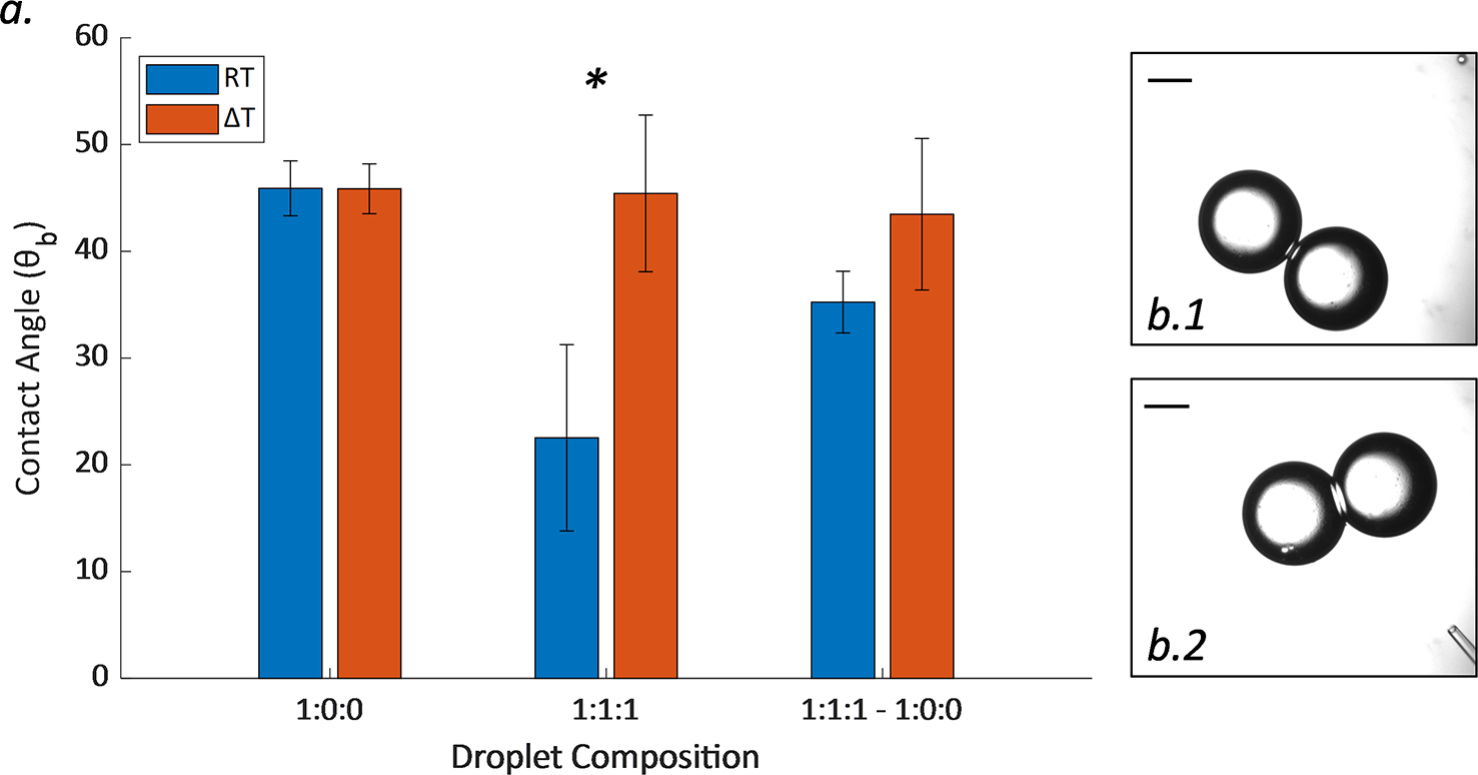
(a) Changes in contact angle for membranes initially at room temperature and after heating and then cooling for 1:0:0 (*n* = 2), 1:1:1 (*n* = 6), and 1:1:1–1:0:0 (*n* = 3) membranes of DPhPC:bSM:cholesterol. The results showed that symmetric distributions of domain-forming lipids produced large changes in the contact angle after heating–cooling (1:1:1 case), where the asterisk (∗) indicates the statistical significance in the comparisons between the room temperature (RT) and heating–cooling cases (Δ*T*). This may be seen in (b), where the membrane dimensions are increased after a heating–cooling cycle. Scale bar represents 250 μm.

Initial experiments investigated the contact angle with the droplets resting in an oil reservoir. Membranes were initially formed without heating the droplets (RT), and each case was heated past the transition temperature and then allowed to cool back down to room temperature again (Δ*T*). A summary of these results may be seen in Figure 7. The 0:1:1 membranes did not form monolayers without heating as shown by their monolayer tension measurements in Figure 6, so they are not included in the data presented here.

A temperature dependence was observed for membranes formed between droplets containing the L_o_ domains, particularly the symmetric distribution cases. Upon heating then cooling, the summed contact angle (θ_b_ = θ_b,1_ + θ_b,2_) between the droplets increased substantially (Figure 7a) and was statistically significant (*p* = 0.0012). This behavior was not significant for cases where the domain-forming lipids were not present in both leaflets (*p* = 0.9904 for 1:0:0, *p* = 0.2035 for asymmetric).

Analysis of these results combined with the pendant drop results for the different lipid mixtures guided the next experimental steps. First, proper monolayer formation may be inhibited by not heating the droplets prior to membrane formation to ensure the suitable packing and insertion of the bSM lipids (Figure 6a, 0:1:1 results). Second, the high standard deviation in the contact angles observed for cases with lipid rafts (Figure 7a, 1:1:1 results) indicated that the properties of the membrane were not static and must be observed over time. The next set of experiments suspend the droplets on two electrodes to measure the membrane electrical properties combined with the energetics. Evolutions of the contact angle over time between the droplets were obtained to investigate the influence of transverse membrane organization on membrane energetics. The contact angle describes the membrane favorability through the energy of adhesion and is presented here as a simple metric for comparison.^45^ The reported contact angle here is the sum of both contact angles (θ_b_ = θ_b,1_ + θ_b,2_).

The symmetric 1:1:1 mixture shows the effects of lipid domain formation on contact angle over time and their influence on the opposing leaflets. These cases exhibited a gradual evolution in the contact angle, increasing far beyond typical measurements for membranes formed in hexadecane^1,36^ and in some cases reaching as high as 80°. The behavior continued for as long as 2 h after formation and appears to occur at a similar time scale to the growth of lipid domains observed by Scheidegger.^46^ A sample video of this evolution with respect to time is provided in Video S1. During the cooling process the membrane had to be adjusted periodically, and direct comparisons should not be made between the Δ*T* and RT membrane formation cases in the first 20 min of the experiments.

**Figure 8:**
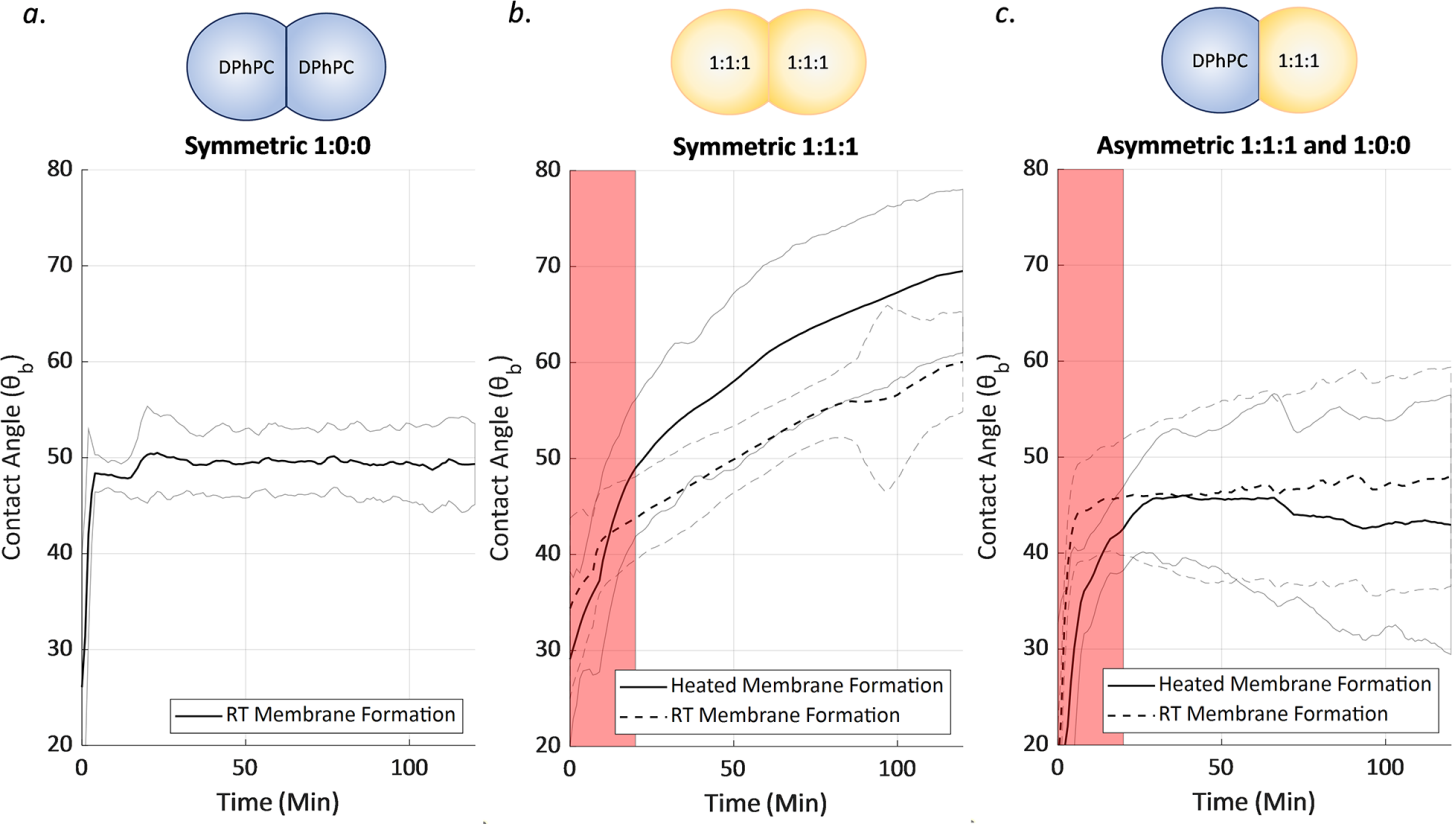
Contact angle measurements over time for varying droplet compositions plotted with the 95% confidence integral. Red panels indicate the cooling period for the heated membrane cases, where direct comparisons should not be made. Subsequent results will typically ignore this region. (a) Symmetric droplet composition of 1:0:0 mixture of DPhPC:bSM:cholesterol (*n* = 3). (b) Symmetric droplet composition of 1:1:1 with the membrane formed at elevated temperature (*n* = 6) and room temperature (*n* = 5) after initial heating. (c) Asymmetric droplet composition of 1:0:0 and 1:1:1 with the membrane formed at elevated temperature (*n* = 3) and room temperature (*n* = 3) after initial heating.

The initial effect is reduced when the membrane is formed after cooling the separated droplets below the transition temperature. Here the two leaflets are brought into contact with domains already established within the leaflets. Cases without the L_o_ domains (Figure 8a) and cases where the L_o_ domains were asymmetrically distributed between the leaflets (Figure 8c) exhibited negligible change in the contact angle over time, suggesting that these gradual shifts are due to registration of the domains as they form within the membrane.

For the asymmetric case presented in Figure 8c there are potential flip-flop exchanges between the two leaflets driven by the membrane asymmetry,^41^ where cholesterol in particular is able to rapidly switch from leaflet to leaflet across the membrane.^47,48^ This has the potential produce a gradual change in the membrane characteristics over time as the asymmetry is reduced but may be counteracted by the tendency of the DPhPC leaflet to exclude cholesterol. In either case, the overall evolution seen in Figure 8b is not present in these asymmetric cases. Furthermore, there is a potential for an electrical offset produced by the asymmetric membrane,^41^ but electrowetting sweeps were not conducted for this study since the electrowetting technique struggled with the gradual evolution of the minimum membrane dimensions.

It is important to note that proper monolayer formation was essential for the phenomena observed in Figure 8b. If the droplets were not heated at any point above the transition temperature (case 3), the gradual growth in the membrane dimensions was not observed over time, and the contact angle remained relatively constant throughout the experiment (Figure S3). This is likely due to the inability of the bSM:cholesterol domains to form the monolayer at room temperature (Figure 6c). Furthermore, if the lipid-buffer solution was not heated during the initial sonication step discussed in the Methods section, the overall trends were severely diminished.

Cases without DPhPC (symmetric 0:1:1) were investigated as well; however, the membranes were prone to coalescence. These were repeated for *n* = 29 times with one successful experiment over the full duration which is presented in Figure S2. Interestingly, this case also exhibited a gradual change in the contact angle, indicating that subdomains are formed with this mixture as well; however, this was not repeatable.

**Figure 9:**
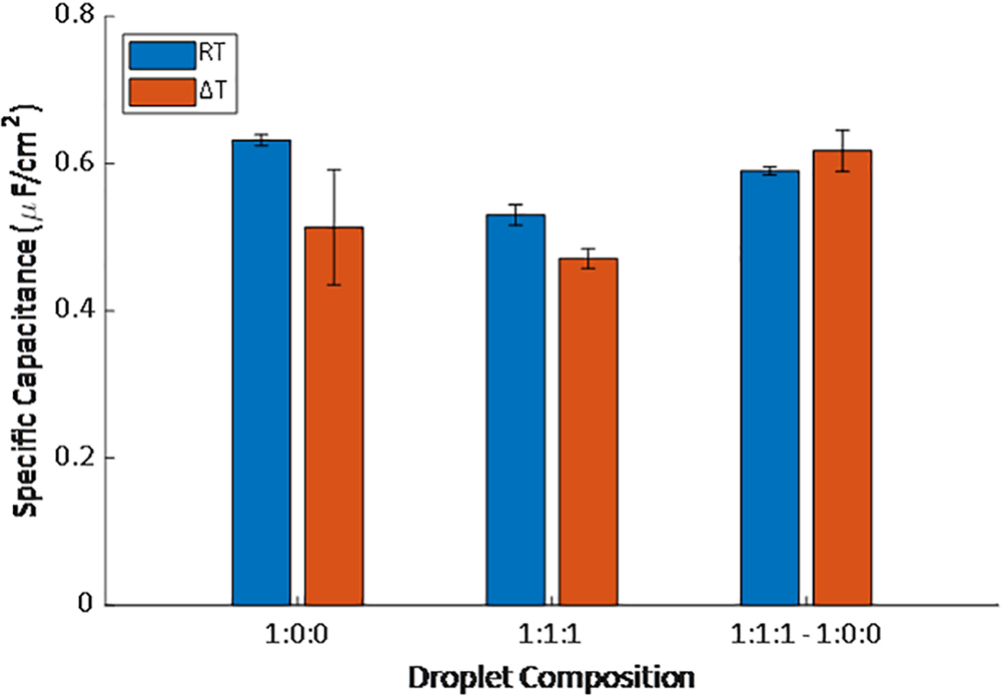
Specific capacitance measurements for various membrane compositions at room temperature with the membrane either formed at elevated temperature (Δ*T*) or room temperature (RT). Membranes containing domains were always heated beforehand to ensure proper monolayer formation.

The specific capacitance was calculated for each case over time. These values were mostly stable over the 2 h duration once the membrane was cooled to room temperature, and their traces over time are provided in Figure S1. The average of the specific capacitance from 20 min onward after the solution reached room temperature is presented in Figure 9. Membranes formed containing L_o_ domains exhibited lower capacitances than membranes from DPhPC alone, indicating greater thicknesses or reduced permittivity.^34^ In this step the specific capacitance for DPhPC alone was reported to vary with the temperature at the membrane formation step—there was a drop in the specific capacitance and the contact angle when the membrane was formed at elevated temperatures (Figure S4). This suggests that there may be trapped residual solvent when the membrane is formed at higher temperatures, reducing the membrane specific capacitance and contact angle, which should be accounted for when examining the 1:1:1 results as well.

**Figure 10:**
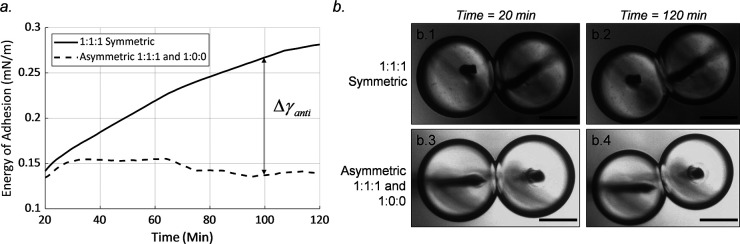
Energy of adhesion reflects the favorability of the membrane. (a) The averaged energy of adhesion for the symmetric and asymmetric membranes after the droplets reached room temperature from cases where the membrane was formed above the transition temperature (*n* = 6 for symmetric, *n* = 3 for asymmetric) shows a clear, gradual improvement. This is further reinforced by (b) the accompanying growth of the membrane as shown in sample images from the experiments, demonstrating the increase in the membrane size after 2 h. The scale bar represents 250 μm.

While the contact angle results in Figure 8 present a general overview of the evolution of the membrane over time, the energy of adhesion presents insights into the mismatch penalty due to antiregistered domains (eq 3). This was calculated for symmetric and asymmetric membranes, both with the membrane formed above the transition temperature, and plotted as shown in Figure 10 after the initial 20 min cooling period to reach room temperature. While steady state was not fully reached even after 2 h, the results show an increase of 0.1–0.2 mN/m in the energy of adhesion for symmetric cases while they both began at a similar point upon cooling to room temperature. This provides estimations of the mismatch penalty associated with this lipid formulation over time.

**Figure 11:**
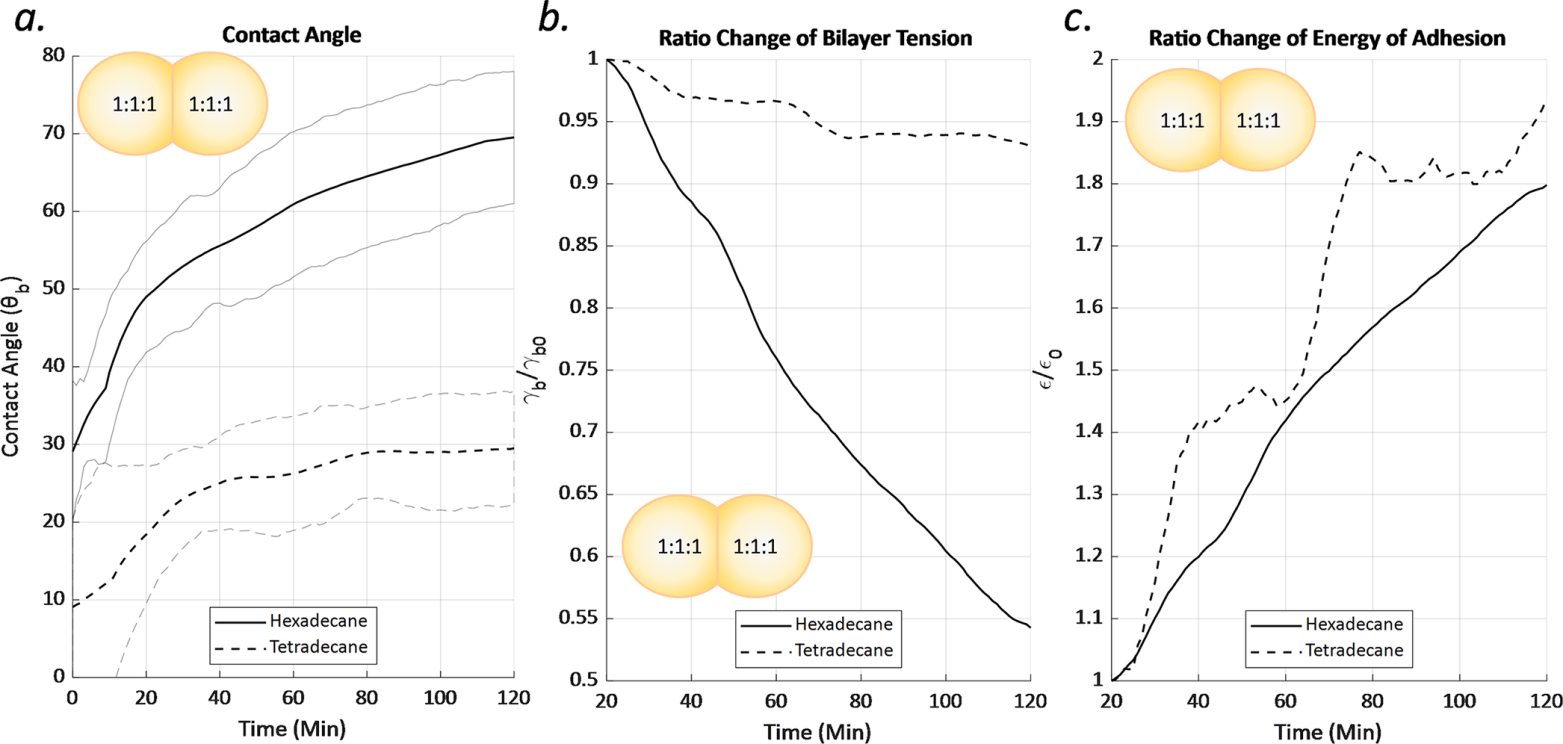
(a) Contact angle for 1:1:1 membranes formed at elevated temperature and then allowed to cool to room temperature over 2 h for membranes formed in hexadecane (*n* = 6) and tetradecane (*n* = 5). The contact angle for hexadecane changes dramatically and produces greater shifts in the relative bilayer tension as shown in (b); however, the relative change in the energy of adhesion for the two mixtures is similar as shown in panel (c). This suggests that residual solvent in the membrane reduces the mismatch penalty, which in turn reduces the rate of change.

Finally, the symmetric experiments with membranes formed above the transition temperature were repeated with tetradecane as the surrounding solvent. Tetradecane produces thicker membranes with greater amounts of residual solvent within the membrane, separating the leaflets and lessening the influence of interdigitation^36,39^ (Figure S1b), reducing the specific capacitance by 0.05 μF/cm^2^. These results showed several trends of interest as summarized in Figure 11. First, the initial contact angle is substantially less for membranes formed in tetradecane, which is in agreement with the literature.^36,39,49^ Second, the change in the contact angle over time is also less pronounced as shown in Figure 11a. If we examine the relative change in the bilayer tension (Figure 11b), the membrane formed with less residual solvent (hexadecane) has a much greater relative change in the bilayer tension over time.

However, if we examine the relative change in the energy of adhesion (Figure 11c), the results for membranes formed in hexadecane and tetradecane converge. This indicates that while the excess residual solvent reduces the interactions between the leaflets across the membrane, it also reduces the mismatch energy penalty associated with antiregistration. The gradual move toward registration is influenced directly by the magnitude of the total penalty associated with antiregistration. The results suggest that the rate of change in the mismatch penalty is equal to its current value, and the membrane will asymptotically approach an optimal arrangement.

## Conclusions

The results from this study are the first demonstration of an evolving DIB membrane influenced by the transverse organization of lipid domains in the two leaflets. Our results showed that the contact angle formed between two droplets containing domain-forming lipid compositions was dependent on the membrane symmetry. Only cases where the lipid domains are present in both leaflets exhibited substantial changes in the bilayer tension over time as the membrane gradually approached registration. Furthermore, cases with the membrane formed with domains already present in the two leaflets exhibited a slower increase in the energy of adhesion relative to membranes formed at temperatures above the transition temperature.

The specific capacitance of the membrane was almost constant for each case, even as the contact angle between the droplets increased. The cases with gradual registration of the domains exhibited smaller values for the specific capacitance, which may be linked to either increases in the membrane thickness or decreases in the relative permittivity of the membrane. Since bSM:cholesterol exhibits both an increased order as well as a longer chain length than DPhPC, this may be a combination of the two factors that cannot be separated experimentally here.

The final experiments observed the influence of residual solvent within the membrane, focusing on the gradual evolution of the bilayer tension and the energy of adhesion. While membranes formed in tetradecane exhibited a much lower change in the bilayer tension alone, the ratio of the energy of adhesion over time presented similar trends to membranes formed using hexadecane. This suggests that the rate of registration is linked to the magnitude of the mismatch penalty. Trapping residual solvent between the lipid leaflets reduces the unfavorable interactions of the antiregistered domains but also reduces the rate of registration addressing these penalties.

In summary, this research investigated the influence of lipid domains in a model membrane system enabling estimations of membrane energetics through drop-shape analysis and monolayer tension measurements. The results indicate that antiregistration of the lipid domains in the leaflets produces a gradual reconfiguration of the DIB membrane, seeking a more optimal, registered configuration over the course of hours. The rate of reconfiguration appears to depend on the energetic penalty associated with antiregistration, and the membrane is likely to asymptotically approach equilibrium. The technique presented here uses the unique liquid-in-liquid structure of DIBs to enable estimations of the mismatch penalty associated with antiregistered domains.

## Supporting Information Available

Additional results for the contact angle, energy of adhesion, and specific capacitance over time (PDF)
Video S1: a representative video of contact angle image processing (AVI)

## Supplementary Material

la4c00958_si_001.pdf

la4c00958_si_002.avi
