# Supplementary material for: Measuring the Transmembrane Registration of Lipid Domains in Droplet Interface Bilayers through Tensiometry
Source: Langmuir. 2024 May 16;40(21):11228–38. doi: 10.1021/acs.langmuir.4c00958 (PMC11140749; doi:10.1021/acs.langmuir.4c00958)
Supplement: Supplementary file 1 — la4c00958_si_001.pdf [file la4c00958_si_001.pdf]

Supplementary Information

Measuring the Transmembrane Registration of Lipid Domains in Droplet Interface Bilayers through Tensiometry

Braydon G. Segars[a], Michelle Makhoul-Mansour[a,b], Joyce El Beyrouthy[a], Eric Freeman[a]*

[a.] School of Environmental, Civil, Agricultural, and Mechanical Engineering, University of Georgia, 110 Riverbend Road, Athens, GA, 30605, United States of America

[b.] Mechanical, Agricultural, Biomedical, and Environmental Engineering Department, Tickle College of Engineering, University of Tennessee Knoxville, 1512 Middle Dr, Knoxville, TN, 37916, United States of America

*ecfreema@uga.edu

Table of Contents

- Additional Results for the Contact Angle, Energy of Adhesion, and Specific Capacitance over Time.
  - Energy of Adhesion and Specific Capacitance for standard cases with respect to time.
  - Energy of Adhesion and Specific Capacitance for 0:1:1 compositions
  - Contact Angle for 1:1:1 compositions with no heating.
  - Contact Angle for 1:0:0 compositions with heating.

Additional Results for the Contact Angle, Energy of Adhesion and the Specific Capacitance over Time

Results for the energy of adhesion and specific capacitance over time are included below. As a reminder, case 1 corresponds to the monolayers and bilayers formed at elevated temperature, case 2 corresponds to monolayers formed at elevated temperature and bilayers formed at room temperature, and case 3 corresponds to no heating of the droplets at any point. The lipids selected for this study are DPhPC:bSM:Chol. Asymmetric membranes are formed between the 1:1:1 and 1:0:0 compositions.

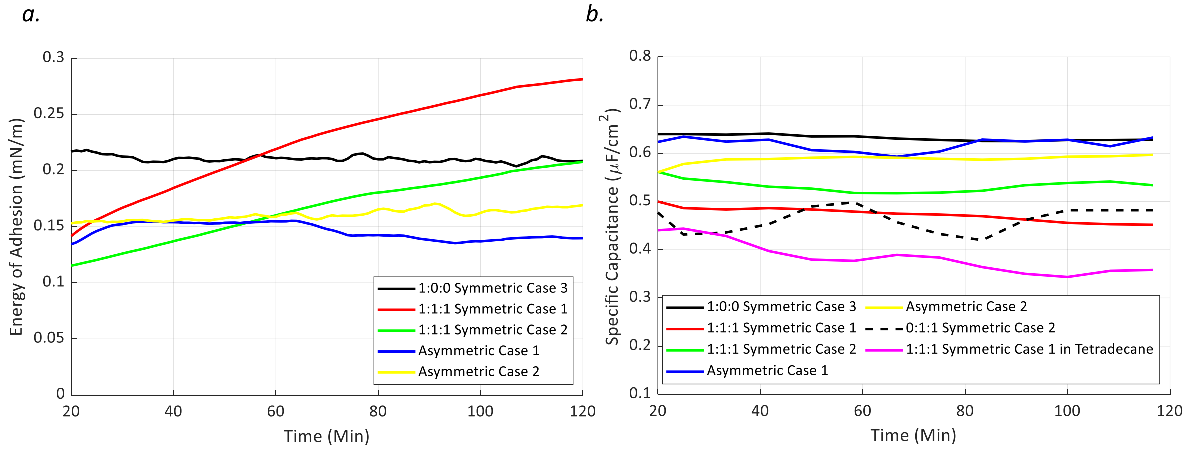

Figure S1 – a) Calculated energy of adhesion over time for all membrane compositions/heating cases and b) the specific capacitance for all cases.

The contact angle and specific capacitance of the 1 successful symmetric 0:1:1 experiment is plotted below as well. The contact angle increased in a similar fashion to membranes formed from the mixtures suggesting an additional ordering behavior may be present where domains are formed within the bSM:cholesterol bilayer; however this experiment was not repeatable after 29 attempts. Future work may establish a reliable method for forming the 0:1:1 membrane.

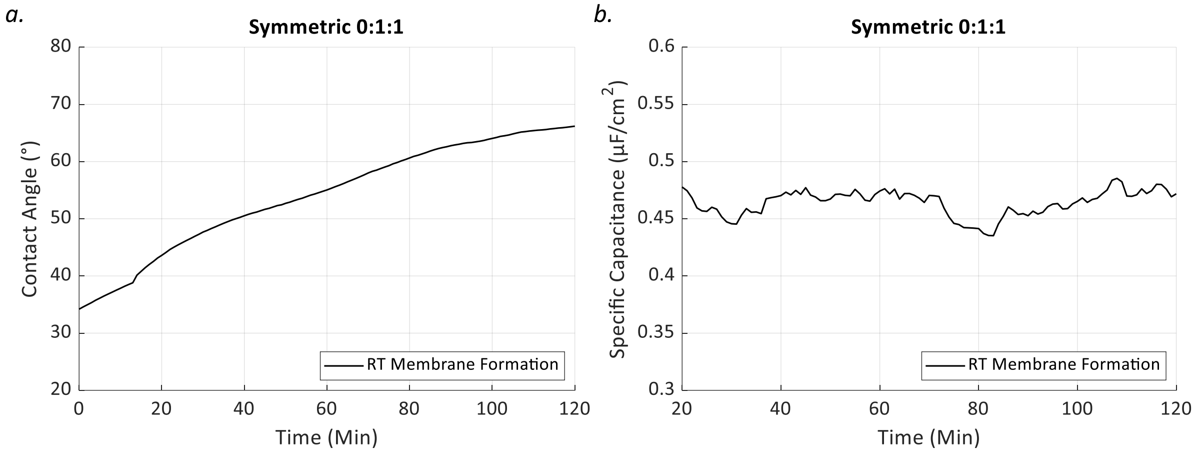

Figure S2 – The 0:1:1 membrane was successfully formed one time over the course of two hours. The results for a) the contact angle showed a similar gradual increase over time in the favorability of the membrane, indicating organization in the bSM:Chol domains as well. b) The specific capacitance appeared to remain generally constant. However, the results could not be replicated after 29 attempts.

When membranes were formed using the symmetric 1:1:1 composition without heating the droplets prior to membrane formation, the contact angle remained approximately constant with larger standard deviations, similar to the asymmetric cases. However, the membrane takes substantially longer to reach equilibrium compared to the 1:0:0 results without heating.

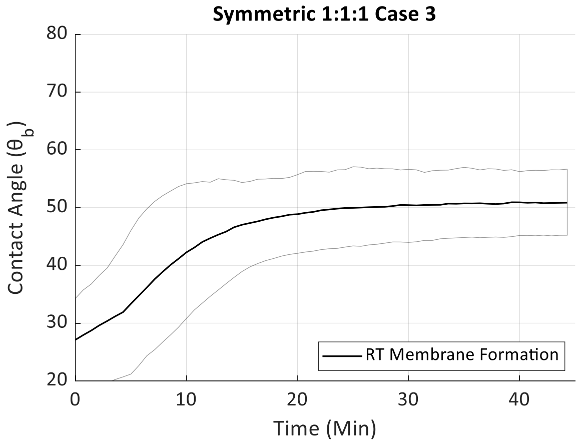

Figure S3 – Forming the monolayers and bilayer at room temperature in the 1:1:1 symmetric case resulted in negligible evolution of contact angle over time (N=4). Plotted with 95% confidence integral.

Membranes formed from DPhPC alone exhibited reduced specific capacitances only when the membrane was formed at elevated temperatures (Case 1). This is potentially due to increased solvent trapped within the membrane, and produced lower contact angles as shown here given the less-favorable membrane formation conditions. Consequently it is expected that the contact angles and specific capacitances

measured for the 1:1:1 symmetric cases formed at elevated temperature may be artificially restricted as well.

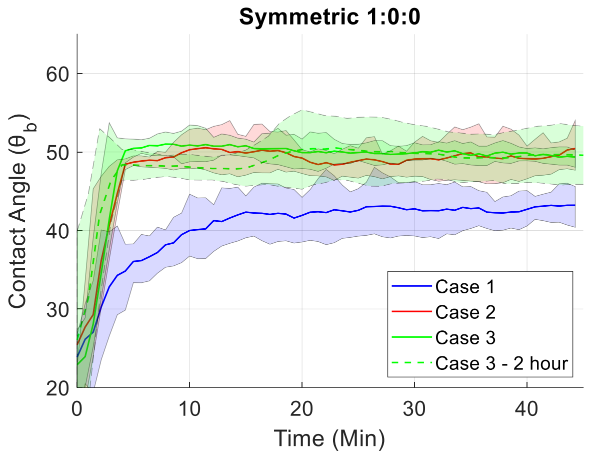

Figure S4 – The 1:0:0 symmetric membranes were formed using all three heating cases. When the membrane is formed at elevated temperature a decrease in the contact angle is observed.
